# Modelling and solving the university course timetabling problem with hybrid teaching considerations

**DOI:** 10.1007/s10951-024-00817-w

**Published:** 2024-10-20

**Authors:** Matthew Davison, Ahmed Kheiri, Konstantinos G. Zografos

**Affiliations:** 1 STOR-i Centre for Doctoral Training, Lancaster University, Lancaster, UK; 2 Department of Management Science, Lancaster University, Lancaster, UK; 3 Department of Management Science, Centre for Transport and Logistics (CENTRAL), Lancaster University, Lancaster, UK

**Keywords:** University timetabling, Hybrid teaching, Binary programming, Multi-objective

## Abstract

The university course timetabling problem is a challenging problem to solve. As universities have evolved, the features of this problem have changed. One emerging feature is hybrid teaching where classes can be taught online, in-person or a combination of both in-person and online. This work presents a multi-objective binary programming model that includes common university timetabling features, identified from the literature, as well as hybrid teaching features. A lexicographic solution method is outlined and computational experiments using benchmark data are used to demonstrate the key aspects of the model and explore trade-offs among the objectives considered. The results of these experiments demonstrate that the model can be used to find demand-driven schedules for universities that include hybrid teaching. They also show how the model could be used to inform practitioners who are involved in strategic decision-making at universities.

## Introduction

Timetabling for universities is a very challenging problem and one of the most interesting educational timetabling problems to research. The interest and challenges arise because the decisions that need to be made impact many different stakeholders and resources. By modelling the university timetabling problem, we hope to gain insight into some of the interactions between many stakeholders and types of resources.

One major factor increasing the difficulty of this problem is that students have a choice in the degree program and a choice in the modules that they attend. This means the output of the university course timetabling problem should be a timetable for each student. This issue is exacerbated by the fact that universities typically enrol significantly more students than other types of educational establishments. Allowing students to choose some of the modules they take means there is a stronger interdependence in these choices. Students can interact with each other and therefore may influence each other’s choices. One possible consequence of this inconsistent class sizes.

Teaching spaces at universities are more diverse than other educational establishments because they are primarily places of research for multiple disciplines, with each discipline requiring specialist equipment and facilities. This means there are more constraints on how classes are assigned to teaching spaces. This diversity contributes to a higher dispersion of facilities. Teaching spaces of different shapes and sizes do not fit together as neatly as teaching spaces that are uniform in size. Therefore, more space is needed for the university which can increase travel time between classes. Dispersion of teaching facilities can also be a consequence of the university layout. Some universities are campus universities meaning most of the buildings are on a single site, but some universities are city universities where university-owned buildings are mixed amongst other buildings. There can be a mix of the two with some universities spanning multiple cities and campuses. Travel time between buildings for any layout needs to be considered when timetabling. Universities also have longer working hours than other educational establishments meaning a greater number of times that classes and other meetings can happen, increasing the scale of the timetabling problem.

An emerging factor that complicates the timetabling process is the increasing use of “hybrid teaching”. This is where a class can be held completely online or in a “hybrid mode” where some students attend in-person and some attend online. The inclusion of hybrid teaching means that student allocation involves deciding what classes a student attends as well as deciding how they attend these classes. Different students respond differently to each mode of teaching and therefore this needs to be accounted for when assigning students to classes.

If a timetable is produced that does not address these complexities, then students and staff will be unhappy with the timetable. Students often pay a lot of money to attend university and a timetable that impacts their ability to attend classes could encourage them to discontinue their studies. For staff who research at the university, a timetable that does not allow them enough time to research around teaching or does not cater to various personal requests could cause them at worst to leave their position at that university, negatively impacting the teaching.

Therefore, being able to produce timetables that can consider the above complexities is imperative to the successful operation of a university. At a high level, universities have strategic goals that they want to achieve (for example, achieving a high output of novel research). This requires careful management of resources and people which automated scheduling can help facilitate. The final timetables produced specify exactly what needs to be done at an operational level to work towards these goals.

There already exists a significant amount of literature that addresses various aspects of the university course timetabling problem. However, to the best of our knowledge, the literature lacks models that explicitly include the emerging issue of hybrid teaching. This paper aims to close this gap by demonstrating how hybrid teaching can be incorporated explicitly into a university timetabling model. This includes specifying what information needs to be known about the university and what sort of variables and constraints need to be part of the mathematical model that produces timetables for students.

The objectives of this paper are to achieve the following: (i) provide a timetabling model that explicitly incorporates hybrid teaching, (ii) identify the benefits of including hybrid teaching in the timetabling model and (iii) discuss how this model gives rise to several interesting research directions and how this model could be used in a strategic decision making context.

The rest of the paper is structured as follows. In Sect. 2, a review of existing work in the context of university timetabling is provided. In Sect. 3, a brief description of the problem to be modelled is given. A mathematical formulation of this problem is given in Sect. 4. The method used to solve this problem is given in Sect. 5 and this method is used in Sect. 6 for various computational experiments. Section 7 includes a discussion about the results and extensions to the model. Finally, in Sect. 8 there is a summary of the work done in this paper.

## Related work

University timetabling problems have been studied for a long time with one of the earliest papers in the literature presenting a method for university examination timetabling (Broder, 1964). There are three types of university timetabling problems: post-enrolment-based timetabling, curriculum-based timetabling and examination timetabling (Lewis and Thompson, 2015).

The examination timetabling problem is the problem of assigning examinations to locations and times. Considerations need to be made to ensure that students can attend all the examinations they need to and to ensure that the locations have enough capacity for the examinations to take place. Curriculum-based timetabling is where events (such as lectures and seminars) are grouped to form fixed curricula which are then assigned to times and locations and post-enrolment-based timetabling is where events are assigned times and locations with respect to student demand and/or enrolment data (Lewis and Thompson, 2015).

This paper focuses on a combination of the curriculum-based and post-enrolment-based timetabling problems known as the university course timetabling problem (UCTTP). The goal of this problem is to assign people to events and these events to times and locations subject to various constraints (Babaei et al., 2015). Events are typically grouped into “courses”, hence the name UCTTP; however, this paper uses the term “module”. The reason we do this is that universities in the UK typically use the phrase “course” to describe a programme of study that is made up of modules. For example, an undergraduate mathematics course may contain modules that cover algebra.

The earliest UCTTP models were graph theoretic models (de Werra, 1985). The university course timetabling is an $\mathcal{N}\mathcal{P}$-hard problem (Cooper and Kingston, 1996) and therefore early mixed integer programming (MIP) models for timetabling problems (see Badri, 1996) could only be solved exactly for small instances. As time went on and universities became bigger and more complex, the demand for sophisticated models and solution methods became greater. Lewis (2008) and Burke et al. (2012) provide reviews that primarily cover heuristic and hyper-heuristic algorithms, approaches that have dominated the field for over two decades. However, thanks to improvements in computers and MIP solvers, matheuristics are the current focus of the timetabling community (see Mikkelsen and Holm, 2022).

There have been several papers reviewing research on the UCTTP. Two recent papers that review some of the state-of-the-art solution methods are Tan et al. (2021) and Chen et al. (2021). For less recent but more feature-focused reviews that are useful for understanding the field see Babaei et al. (2015) and Aziz and Aizam (2018).

### Key timetabling features

In this section, papers are reviewed according to general model features that are of importance when tackling the UCTTP. For each paper, a brief description of the paper is given and what it contributes to the literature is highlighted. Model features, the modelling approach, and the data used are summarised for each paper in Tables 1, 2 and 3.

#### Typical resource allocation

Badri (1996) proposes a binary program to model a departmental assignment problem at the United Arab Emirates University. This paper, to our knowledge, is the earliest paper to include constraints similar to the set-packing problem (Skiena, 2008) which are useful when you need to constrain choosing a certain number of items selected from a collection of options. In their model, they penalise using more rooms than available and not meeting instructor preferences. This is done using a goal programming approach where there are penalties for deviations above or below a desired level.

Di Gaspero and Schaerf (2006) use a local search method to solve the course timetabling problem involving assigning lectures for courses to periods and rooms. The main purpose of their paper is to demonstrate their local search method. By using a simple solution representation, moves between solutions in the search do not lead to infeasibility. The two hard constraints maintained between moves are ensuring no more than one lecture happens in a single room at the same time and ensuring all lectures in a module are offered. The quality of the solution is a weighted sum of violations of soft constraints. The soft constraints include features such as room capacity and instructor availability. They also include temporal constraints spreading lectures across several days and spacing lectures within days to avoid gaps.

Overlapping timeslots and irregular weekly timetables are allowed in the problem defined by De Causmaecker et al. (2009). This was to accommodate the structure of teaching at KaHo Sint-Lieven School of Engineering. A feature not included in their problem that others such as Badri (1996) include is the assignment of staff. It is assumed staff already know what they will teach so constraints are included to ensure they can attend all events they need to. The solution method is very similar to Di Gaspero and Schaerf (2006), using a local search algorithm to find solutions.

Chaudhuri and De (2010) define a timetabling problem including many of the features seen in the problems discussed so far. This includes various temporal constraints, staff preferences and conflicting resource assignments. A constraint seen in this problem that has not been discussed yet is ensuring that assignments are “compatible”. For example, a chemistry class may only be held in a chemistry laboratory and so the set of compatible rooms for a chemistry class is the set of all chemistry laboratories at the university. This notion of compatibility extends to any assignment of events or people to resources.

The problem described in Aizam and Caccetta (2014) is a binary program like in Badri (1996). In this paper, they start by describing a basic model that contains constraints that they deem necessary for every timetabling problem and then suggest extra constraints to account for additional features that could be included. This is one advantage of using a binary program formulation. One feature in this model (and the previously discussed models) that is worth pointing out is “completeness”. This is where every event is assigned resources or every student is assigned to every class they need to be.

An example of a model where completeness is not necessary is given by Méndez-Díaz et al. (2016). There is more emphasis on the post-enrolment features of the UCTTP. The objective of their model is to maximise the total weighted preference for the assignments of students to modules. Due to student demand driving the timetable, it is not necessary for all events to be assigned a location and a time. One feature that causes this uncertainty in whether events are assigned or not is the structure of modules. In Méndez-Díaz et al. (2016), modules are composed of one or several commissions, which are instances of the same module. The literature also refers to this as “configurations” (Müller et al., 2018). If it is known that all students are assigned to a single commission, then the events in other commissions do not need to be assigned.

The Integer Linear Program (ILP) described by Fonseca et al. (2017) covers many of the features described in the models seen so far. It includes some of the constraints outlined by Aizam and Caccetta (2014) in their basic model and also includes constraints described by the eXtended Markup Language for High School Timetabling (XHSTT) format (Post et al., 2014). This format is one example of an attempt to generalise a description of any high school timetabling problem.

#### Scheduling issues

One of the earlier discussions surrounding student scheduling issues is given in Carter (2001). This model operates a “demand driven” approach where students choose modules and then a timetable is found to best match these requests. They use clustering techniques to group students with similar requests and assign these to sections to minimise expected conflicts. Then once a timetable is found, the student sectioning is repeated considering individual student conflicts.

In the model described by Schimmelpfeng and Helber (2007), room assignment and staff assignment are the most important features. Rooms should not be assigned to more than one class at a time or contain classes with an attendance that exceeds the room capacity. Staff members can not be assigned to a time when they are not available and other staff preferences should be respected. These include a variety of teaching staff requests such as breaks, consecutive or distributed teaching slots, and a maximum number of teaching slots. Unlike the timetabling seen in other papers such as Carter (2001), students are an afterthought.

Gonzalez et al. (2018) create a MIP that schedules courses for the United States Air Force Academy (USAFA). The interesting scheduling issue in this problem is that students who are at the USAFA have work commitments as well as academic commitments. They utilise a goal programming approach to meet as many requirements as possible including minimising student registration conflicts, where a student is assigned two modules that conflict. They state that in practice it is impossible to remove every conflict.

The MIP model described by Holm et al. (2020) was constructed to solve the problem designed by the organisers of the ITC-2019 (Müller et al., 2018). In this problem, student conflicts are minimised as part of a weighted objective. In their follow-up paper, where they describe their graph-based MIP (Holm et al., 2022), they discuss the importance of identifying assignments that lead to inevitable conflicts and assignments that lead to impossible conflicts. This preprocessing step helps minimise the work that needs to be performed by the solution method.

#### Student movement and travel

When dealing with the movement of students, one aspect (Daskalaki and Birbas, 2005) aim to control is the number of classroom changeovers. Minimising the number of classroom changes means that there is less noise and congestion in spaces on campus. To do this, they name a preferred classroom for each student group and try to ensure that the group stays in that classroom and has consecutive sessions in that room. This is however not a realistic representation of a general university as students cannot typically be grouped so easily and need to be considered as individuals.

Al-Yakoob and Sherali (2007) deal with parking and traffic congestion issues in their paper. This is achieved primarily by limiting the number of students on campus at any given time. A hard limit for the whole campus could lead to some timeslots having a few very crowded departments whilst the others are empty. This is unfair to the busy departments so to make this fair they also impose a minimum and maximum attendance at the department level to distribute congestion over the entire campus.

Vermuyten et al. (2016) also try to avoid congestion as in Daskalaki and Birbas (2005), however, their approach does not try to achieve this by fixing students in one place but by changing how many students move along various corridors at a given time. A graph that represents the faculty building is used so they can optimise the flow of students through arcs and the resulting travel times. The element they minimise overall is the maximum travel time seen in an arc. A two-stage decomposition is used where most of the schedule is determined in the first stage, and classes and rooms are swapped around in the second to locally optimise student flow. Optimising the schedule and the flow together is computationally expensive.

Gogos et al. (2022) work with a problem that focuses on minimising the number of times in a week that students travel to university. The motivation is that students who do not live on campus do not want to spend excessive money on public transport and want to reduce the risk of catching an illness from other passengers. They approach this problem by calculating the minimum number of days a student would need to attend university and then trying to minimise the number of excess days the student is on campus. This is limited as it does not consider the time of travel (certain times are busier) or if students make multiple journeys in a single day (multiple campuses).

#### Scarce resources

The timetabling model in Dammak et al. (2008) includes a few of the features seen in other papers. One feature relating to the usage of resources is their aim of maximising the occupancy of classroom seats. Since their paper presents only a heuristic to produce a feasible solution, this objective is not explicitly optimised. However, in the construction of the feasible solution, they order the classrooms and student groups in a non-increasing fashion so that large student groups are placed in large classrooms.

Lindahl et al. (2018) approach the UCTTP differently. They break from the operational timetabling problem and move towards a strategic approach. Three problems are presented in this paper. The first is the “quality problem” that is similar to the other papers that produce a timetable that is high quality by some measure. The second finds the minimum number of rooms needed. The third finds the minimum number of times needed. They solve a collection of bi-objective models to create solution frontiers that can be used to analyse gain in quality by not using the minimum amount of resources.

Barnhart et al. (2022) experience scarce resources due to the COVID-19 pandemic. This context applied to most if not all universities at the time. They tackle a term-planning problem and a timetabling problem within the same paper. The timetabling problem involves working out when and where events take place except for modelling purposes classrooms are bundled into blocks. These blocks can be considered as “larger classrooms” but there are fewer of them in total. The idea at MIT was to have students “rotate” between coming onto campus and attending online. Like the work of Al-Yakoob and Sherali (2007), they have a global cap on the number of students on campus at any time to reduce the usage of unscheduled resources (toilets and shops, for example).

#### Hybrid teaching

The only paper, to our knowledge, that explicitly discusses the timetabling problem with hybrid teaching is Barnhart et al. (2022). In this paper, the online teaching space is modelled as a fictitious block of classrooms with zero in-person capacity. The timetabling model tries to maximise the number of modules students can attend with a preference for the in-person format. However, the limitation of this model is that classes can only be offered online or in-person rather than potentially having some students attend physically and some attend online.

An example of where we can see multiple instructional modes, including a true hybrid approach, is in the open-source solver UniTime (UniTime, 2023). These features are implicit here and in the description of the ITC-2019 problem (Müller et al., 2018) as the ITC-2019 problem is a simplified variant of the UniTime problem. For example, the ITC-2019 problem may have two classes that should occur simultaneously with one class not requiring a room assignment, emulating a hybrid setup. There are also cases where classes do not require rooms or where the class subscription limit is greater than the capacity of all the available rooms.

### Our contributions

This review of the literature outlines not only the importance and continued relevance of the UCTTP, but also outlines some of the features of the problem. These include features that are very common across models as well as features relating to specific or emerging issues. Features seen in the literature have been collected in Table 2. Table 3 provides information on how the problem was modelled and what data was used. For both tables, the columns have been ordered by year of publishing.

Table 2 shows that the choice of features included varies from model to model. This is because authors have tried to take on the timetabling issues present at the university where they work. The result is that much of the literature consists of very focused models that do not generalise well, implicitly seen in Table 3 where 76% of papers in this review use internal data to solve the problem.

Table 2 shows that the most studied features include room capacity issues, room and time preferences and staff/student conflicts. Table 3 suggests that the most popular modelling approaches include integer/binary programs. Table 2 is also useful for spotting emerging features of interest. The most notable aspect is the increasing number of models that are primarily driven by student demand or models that consider individual student requests.

It can also be seen from Table 2 that as the field has progressed over time, researchers are generally including more features in their models. This is also reflected in Table 3 which shows that researchers and practitioners in the timetabling field are starting to explore bi-objective and multi-objective approaches.

One major gap in the research is the explicit study of online or hybrid teaching. During the COVID-19 pandemic, many universities needed to adapt to using these formats. However, the university timetabling problem with hybrid module delivery considerations has not been adequately addressed. The model that is presented in this paper includes “traditional” features of the UCTTP and explicitly incorporates the new element of hybrid teaching. The aim of this is to introduce one approach to explicitly modelling hybrid teaching at universities using binary programming so that other researchers can include hybrid teaching in future models. Due to the rarity of this feature in existing models, there is little analysis of how this feature impacts other features of the timetabling problem that are well-studied.

Students and staff at universities are acutely aware of the pedagogical and logistical issues relating to hybrid teaching and the starting point for resolving these issues is being able to represent hybrid teaching in a mathematical sense. With this in mind, the key contributions of this paper are the following:

- Outline the information that needs to be collected about the classes to determine if a class can happen in the online/hybrid mode and what extra information about students needs to be known to best cater to their teaching preferences.
- Present a description of a generic UCTTP with hybrid elements along with a proposed binary program formulation of the problem.
- Demonstrate the model using the most up-to-date benchmark data available to show how the hybrid elements would work in practice and illustrate some of the interactions with other problem features.
- Discuss how this model could be used to produce a timetable that achieves a particular strategic goal as well as identify unresolved logistical problems with hybrid teaching and the research questions that these problems present.

**Table 1:** Indices of the features, modelling approaches and data referenced in Tables 2 and 3

	Index	Description
Features	1	Conflicts for teaching staff are considered
	2	Conflicts for students are considered
	3	Complete timetable
	4	Overlapping times
	5	Explicit use of online classes
	6	Explicit use of hybrid classes
	7	Mode requests
	8	Student choice in modules
	9	Students have compulsory modules
	10	Staff travel time considered
	11	Student travel time considered
	12	Room capacity
	13	Co/Prerequisite courses
	14	Individual student assignment
	15	Room assignment restrictions/preferences
	16	Time assignment restrictions/preferences
	17	Number of students on campus limited
	18	Enrolment data used in the model
	19	Physical student flows are considered
	20	Switching classes or locations
	21	Rooms and equipment have capacity and usage restrictions
	22	Compact timetable preferred
Modelling approaches	1	Mixed integer program
	2	Integer program
	3	Binary program
	4	Neighbourhoods
	5	Graph colouring
	6	No explicit objective
	7	Single objective
	8	Bi-objective
	9	Multi-objective
Data used	1	Institution (Data from the author’s university)
	2	International Timetabling Competition 2019 (Müller et al., 2018)
	3	International Timetabling Competition 2011 (Post et al., 2013)
	4	International Timetabling Competition 2007 (Mccollum et al., 2010)

**Table 2:** Summary of what features are considered by a paper. Feature numbers are given in Table 1

References	Feature																					
	1	2	3	4	5	6	7	8	9	10	11	12	13	14	15	16	17	18	19	20	21	22
Badri (1996)			✓									✓				✓						
Carter (2001)	✓	✓						✓	✓			✓		✓	✓	✓		✓			✓	
Daskalaki et al. (2004)	✓	✓	✓									✓			✓	✓			✓	✓	✓	✓
Avella and Vasil’Ev (2005)	✓	✓							✓			✓			✓	✓				✓	✓	✓
Di Gaspero and Schaerf (2006)	✓	✓	✓						✓			✓			✓	✓					✓	✓
Schimmelpfeng and Helber (2007)	✓	✓										✓	✓			✓					✓	
Al-Yakoob and Sherali (2007)			✓									✓			✓	✓	✓				✓	✓
Dammak et al. (2008)	✓	✓	✓									✓			✓	✓					✓	✓
De Causmaecker et al. (2009)	✓	✓	✓	✓								✓			✓	✓					✓	
Chaudhuri and De (2010)	✓														✓	✓			✓			✓
Santos et al. (2012)	✓	✓	✓													✓						✓
Aizam and Caccetta (2014)	✓	✓	✓						✓			✓	✓			✓					✓	✓
Méndez-Díaz et al. (2016)	✓	✓						✓	✓			✓		✓	✓	✓		✓			✓	✓
Vermuyten et al. (2016)		✓	✓						✓	✓	✓	✓			✓	✓			✓	✓	✓	✓
Fonseca et al. (2017)	✓	✓							✓			✓	✓		✓	✓					✓	✓
Lindahl et al. (2018)	✓	✓	✓									✓			✓	✓					✓	✓
Gonzalez et al. (2018)	✓	✓							✓			✓		✓	✓	✓		✓				
Barnhart et al. (2022)		✓			✓		✓	✓	✓			✓	✓	✓	✓	✓	✓	✓			✓	✓
Holm et al. (2022)	✓	✓	✓	✓				✓		✓	✓	✓	✓	✓	✓	✓		✓			✓	
Gogos et al. (2022)		✓	✓					✓	✓					✓		✓		✓		✓		✓
This paper	✓	✓		✓	✓	✓	✓	✓	✓	✓	✓	✓	✓	✓	✓	✓		✓			✓

**Table 3:** Summary of what modelling approach and data a paper uses

References	Modelling approach									Data used			
	1	2	3	4	5	6	7	8	9	1	2	3	4
Badri (1996)			✓						✓	✓			
Carter (2001)					✓		✓			✓			
Daskalaki et al. (2004)		✓					✓			✓			
Avella and Vasil’Ev (2005)		✓					✓			✓			
Di Gaspero and Schaerf (2006)				✓			✓			✓			
Schimmelpfeng and Helber (2007)		✓					✓			✓			
Al-Yakoob and Sherali (2007)	✓						✓			✓			
Dammak et al. (2008)			✓			✓				✓			
De Causmaecker et al. (2009)				✓			✓			✓			✓
Chaudhuri and De (2010)						✓				✓			
Santos et al. (2012)	✓						✓						
Aizam and Caccetta (2014)		✓					✓			✓			✓
Méndez-Díaz et al. (2016)			✓				✓			✓			
Vermuyten et al. (2016)		✓					✓			✓			✓
Fonseca et al. (2017)		✓					✓					✓	
Lindahl et al. (2018)	✓							✓					✓
Gonzalez et al. (2018)	✓					✓				✓			
Barnhart et al. (2022)		✓							✓	✓			
Holm et al. (2022)	✓				✓		✓				✓		
Gogos et al. (2022)	✓	✓					✓			✓			
Our model		✓	✓						✓		✓		

Modelling approach and data numbers are given in Table 1

## Problem description

What our review has exposed is a lack of literature explicitly modelling hybrid teaching at universities. Where there are explicit instances, there is a lack of analysis regarding the benefits or drawbacks associated with the incorporation of this feature. As stated in the previous sections, the objective of this paper is to focus on this particular feature of the UCTTP and provide some managerial insights regarding this feature. This feature is important to be studied in conjunction with other features of the timetabling problem to shed light on its impact on the resulting schedule. In this section, we introduce a multi-objective university timetabling model with hybrid teaching considerations.

The timetabling problem we are modelling is the post-enrolment approach where students provisionally select modules they want to take and after this is done, a timetable is constructed. Timetabling practitioners at universities in the UK often take this approach. This timetable is typically constructed before the term starts, especially in the case of the first term when new students register in August and start studies in late September. To mirror this process, the timetabling problem in this paper is also a post-enrolment timetabling problem. Part of the input to the mathematical model is a list of students and modules they are requesting to take. A module request is met if the student is assigned to an appropriate arrangement of classes (the particular arrangement varies between universities).

As we are focusing on hybrid teaching, an additional input is that each student may also provide a preference for a particular mode of module delivery (online and/or in-person). If a student expresses a preference for a particular mode then it is assumed this preference applies to all modules they want to attend.

One novel element here is that the travel time between two classes may be different for two students taking the same classes but in different modes. For example, a student attending online only can in theory switch instantly between classes whereas a student attending in-person will need to walk between rooms. If a student does not have enough time to travel between classes or is assigned classes that overlap, this is referred to as a scheduling issue.

The decisions we are making in this problem are the following: (i) when and where are classes being held, with the option for classes to be held online and in-person simultaneously and (ii) what classes students attend and the mode of study they attend the classes. These decisions are made with respect to three different objectives: (i) maximising module requests met, (ii) minimising the total number of scheduling issues and (iii) minimising the total number of classes where a student does not attend in their preferred mode.

There are constraints on these decisions. Classes are only assigned to compatible times. What makes a time compatible for a class depends on the university, however, what we mean by compatible is that the time meets some set of criteria that allows the class to be assigned to it. Similarly, classes are only assigned to spaces that are available and compatible. In this case, available means that the space is not in use by other classes and is free to be used by a class. For example, a chemistry class may need to occur in the afternoon to allow for the setup of equipment in the morning and cannot be assigned a space without the correct equipment or space in use by people doing a different experiment.

Hybrid teaching is only done if the class is assigned the appropriate space. For a teaching space to be capable of hosting a hybrid meeting, it needs to have a particular layout and equipment. In practice, it is not usual that every room meets these criteria. Therefore the collection of physical spaces at the university can be partitioned into those that are capable of hybrid teaching and those that are not capable of hybrid teaching. There are two limits on class attendance. There are limits imposed on the number of students attending a class for pedagogical reasons and room capacity limits so that students can fit into the physical space assigned to the class.

The structure of modules is assumed to be the same as in the ITC-2019 competition (Müller et al., 2018). Modules are made up of configurations, which are made up of subparts. Each subpart contains a collection of classes. For a student to attend a module, they need to attend a class from every subpart within a single configuration. For example, a module may have one configuration containing two subparts. The first subpart could contain a single class in the form of a lecture. The second subpart could contain multiple classes that are seminars. This structure is used in this problem for two reasons. Firstly, it is a good representation of most forms of university modules. Secondly, it means it is easier to utilise the ITC-2019 data sets for testing. The modelling of teachers and instructors is also done in a fashion similar to the ITC-2019 competition where we ensure that a staff member can attend some list of classes without any scheduling issues (no overlaps/sufficient travel time). These lists of classes are another input to the model. This is adopted here for the same reasons we adopt the module structure.

Modules at UK universities are either compulsory or optional (elective). Compulsory modules are those that students are required to take, and elective modules are ones that the student can choose. Table 2 shows that several models in the literature also include this feature. In the problem described here, students are assigned to their compulsory modules as a hard constraint and the number of elective modules that can be attended is maximised. The total number of modules that a student attends is capped at a fixed number.

To summarise, we have designed a variant of the UCTTP that includes hybrid teaching. This variant contains objectives and constraints often referred to as “essential constraints” (Sørensen and Dahms, 2014; Aziz and Aizam, 2018; Rudová et al., 2011) that have been modified to explicitly include hybrid teaching.

The model is designed this way to maintain focus on the novel aspect of modelling hybrid teaching explicitly whilst including key elements of a typical UCTTP formulation for these novel features to interact with.

Before providing the mathematical formulation of the problem, it is important to note that there are some important university features not included in this model that are included in other models such as the model described by the ITC-2019 competition (Müller et al., 2018). In that model description, there are “distribution constraints” that enforce rules on how classes should be distributed in the schedule (for example, ensuring certain groups of classes happen on different days or in the same room). These are not included in this model but could be modelled using the notation provided in this paper to bring the problem even closer to the real-life problem (see Holm et al., 2022).

## Mathematical formulation

Before detailing the mathematical formulation specific, terminology and sets are introduced to make the presentation of the model more efficient. Firstly, the main variables are defined. Secondly, the objectives to be optimised are defined. Finally, it is explained how these objectives are constrained.

### Terminology and notation

#### Timeslots and timesets

**Fig. 1:**
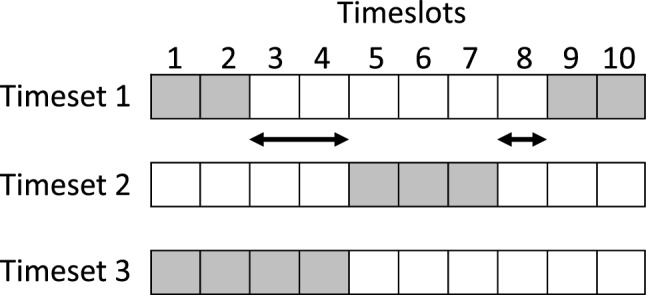
An illustration of a situation where there are ten timeslots and three timesets. The arrows show the “gaps” between the timesets one and two, with the minimum distance “between” the timesets being one timeslot. The intersection of timesets one and three is the set containing timeslots one and two, this means that timesets one and three overlap

In this model, it is assumed that the university term is split into equal lengths of time called timeslots, and a timeset is defined as a subset of these timeslots. This allows for complicated arrangements to be described. For example, an arrangement where a class occurs every other week of term and starts at 9:30 on Mondays can be described by a single timeset. We say that two timesets overlap if the intersection of these sets is non-empty. We define the time between two timesets as the minimum number of timeslots between any two timeslots. Figure 1 illustrates these definitions.

#### Set definitions

The following list outlines the definitions of the sets that are used in the mathematical formulation of the model. In a slight abuse of notation, we use the set *G* as a placeholder for another set that would be a subset of some larger set. This is to streamline some of the definitions by avoiding repetition.

*S*: Set of students.
*H*: Set of teaching staff.
*C*: Set of classes.
*K*: Set of modules.
*L*: Set of timeslots.
*T*: Set of timesets.
*R*: Set of spaces where classes can occur.
$$K_s$$:
Set of modules requested by student $s \in S$. $K_s \subseteq K$.
$$K_s^\text {core}$$
: Set of compulsory modules student $s \in S$ is required to attend. $K_s^\text {core} \subseteq K$.
$$K_s^\text {elec}$$
: Set of elective modules student $s \in S$ would like to attend. $K_s^\text {elec} \subseteq K$.
$$S_k$$:
Set of students requesting module $k \in K$. $S_k \subseteq S$.
$$R_{r}^{u}$$:
Set of timeslots when room $r \in R$ is unavailable. $R_{r}^{u} \subseteq L$.
$$R_c$$:
Set of spaces that are suitable for class $c \in C$ to use. $R_c \subseteq R$.
$$T_c$$:
Set of timesets that are suitable for class $c \in C$ to use. $T_c \subseteq T$.
$$R_G$$:
Let $G \subseteq C$. The set $R_G$ is defined as $R_G:= \cap _{c \in G} R_c$.
$$C_G$$:
Let $G \subseteq C$. The set $C_G$ is defined as $C_G:= \{(c_1,c_2) \in G \times G: c_1 \ne c_2 \}$.
$$R_r^C$$:
Let $r \in R$. The set $R_r^C$ is defined as $R_r^C:= \{ c \in C: r \in R_c\}$.
$$O_l$$:
Let $l \in L$. The set $O_l$ is defined as $O_l:= \{t \in T: l \in t\}$.
$$F_k$$:
Set of configurations for module $k \in K$.
$$P_{f,k}$$:
Set of subparts for configuration $f \in F_k$, where $k \in K$.
$$C_{p,f,k}$$:
Set of classes for subpart $p \in P_{f,k}$, where $f \in F_k$, where $k \in K$.
$$C_s$$:
Set of classes that student $s \in S$ could take if offered. In particular, $C_s = \cup _{k \in K_s}\cup _{f \in F_k}\cup _{p \in P_{f,k}} C_{p,f,k}$.
$$C_h$$:
Set of classes that teaching staff member $h \in H$ must attend if offered.

#### Travel time

Define *A* as the matrix with entries that approximate the travel time between pairs of rooms. In particular, for two rooms $r_1,r_2 \in R$, the entry $A_{r_1,r_2}$ is equal to the number of timeslots that it takes to travel from $r_1$ to $r_2$. It is assumed that *A* is symmetric and that $A_{r,r} = 0$.

#### Hybrid teaching elements

Physical rooms have a finite capacity. For a room $r \in R$, the capacity is denoted as *cap*(*r*). The online space is modelled as a room that is always available and can host multiple classes at the same time. The capacity of this space is considered unlimited, that is, $cap(r^*) = \infty$. This space will be denoted as $r^*$ and a class $c \in C$ can be held online if and only if $r^*\in R_c$. For pedagogical reasons, classes still have a subscription limit, denoted as *sub*(*c*) for $c \in C$. This subscription limit is the maximum number of students who can take a particular class.

It is assumed that students can move instantly from an online class to an online class and that it is a fixed number of timeslots $d^*\in \mathbb {Z}^+_0$ to travel from an online class to an in-person class and vice-versa. In particular, $A_{r^*,r^*} = 0$ and $A_{r^*,r} = d^*$ for all $r \in R{\setminus } \{r^*\}$.

Classes are allowed to be taught in a hybrid format provided that the physical room has the proper equipment. Define $R^h$ as a subset of *R* containing all of the locations that allow for hybrid teaching. For a class to be a hybrid class then not only does the room assigned to the class need to be in $R^h$ but the online portion of the class needs to be scheduled for the same time as the in-person class. For consistency, $r^*\in R^h$.

Different students prefer different modes of teaching, or may not have a preference. Define $\pi _s$ as the preference of student $s \in S$. $\pi _s$ is equal to one if the student prefers in-person teaching, negative one if the student prefers online teaching, and zero if they have no preference towards either format.

#### Module restrictions

Students should not take an excessive number of modules for both financial and pedagogical reasons. We introduce a parameter $k^\text {cap}_s$ that indicates the maximum number of modules student $s \in S$ is allowed to take.

#### Parameter arrays

To streamline the discussion of constraints in the timetabling, the notion of a parameter array is introduced. These are fully determined by the input data and record various relationships between timesets and rooms. The following list provides the definitions of these parameter arrays.

$$D_0$$:
A matrix where $D_0[r,t]$ is equal to one if room *r* is unavailable at some point during timeset *t*, zero otherwise.
$$D_1$$:
A matrix where $D_1[t_1,t_2]$ is equal to one if $t_1$ overlaps $t_2$, zero otherwise.
$$D_2$$:
An array where $D_2[r_1,r_2,t_1,t_2]$ is equal to one if there is not enough time between $t_1$ and $t_2$ to travel between $r_1$ and $r_2$, zero otherwise.

### Variables

$$x_{c,r,t}$$:
Binary decision variable indicating if class $c \in C$ is held in space $r \in R$ during timeset $t \in T$.
$$y_{c,r}^R$$:
Binary decision variable indicating if class $c \in C$ is held in space $r \in R$.
$$y_{c,t}^T$$:
Binary decision variable indicating if class $c \in C$ is held during timeset $t \in T$.
$$g_k$$:
Binary decision variable indicating if module $k \in K$ is offered.
$$q_{k,f}$$:
Binary decision variable indicating if configuration $f \in F_k$ of module $k \in K$ is offered.
$$w_{k,f,p}$$:
Binary decision variable indicating if subpart $p \in P_{f,k}$ in configuration $f \in F_k$ of module $k \in K$ is offered.
$$a_{s,k,f,p,c}$$:
Binary decision variable indicating if student $s \in S$ is assigned class $c \in C_{p,f,k}$, where $p \in P_{f,k}$, where $f \in F_k$, where $k \in K$.
$$\alpha ^{\text {onl}}_{s,c}$$:
Binary decision variable indicating if student $s \in S$ is assigned to the online version of class $c \in C$.
$$\alpha ^{\text {inp}}_{s,c}$$:
Binary decision variable indicating if student $s \in S$ is assigned to the in-person version of class $c \in C$.
$$b_{s,k,f,p}$$:
Binary decision variable indicating if student $s \in S$ is assigned some class in subpart $p \in P_{f,k}$, where $f \in F_k$, where $k \in K$.
$$m_{s,k,f}$$:
Binary decision variable indicating if student $s \in S$ is assigned to configuration $f \in F_k$, where $k \in K$.
$$n_{s,k}$$:
Binary decision variable indicating if student $s \in S$ is assigned to module $k \in K$.
$$\beta ^{\text {onl}}_{s,c,t}$$:
Binary decision variable indicating if student $s \in S$ attends class $c \in C$ during timeset $t \in T$ in the online format.
$$\beta ^{\text {inp}}_{s,c,t}$$:
Binary decision variable indicating if student $s \in S$ attends class $c \in C$ during timeset $t \in T$ in the in-person format.
$$\gamma _{s,c,r,t}$$:
Binary decision variable indicating if student $s \in S$ attends class $c \in C$ in room $r \in R$ during timeset $t \in T$.
$$\tau _{s,c}$$:
Binary decision variable indicating if student $s \in S$ is not attending class $c \in C$ in their preferred mode.
$$h_{s,c_1,c_2}$$:
Binary decision variable indicating if there is a scheduling issue with assigning student *s* to $c_1 \in C$ and $c_2 \in C$.

### Objectives

#### Maximise the total number of elective module requests met

Individual students provide a list of elective modules they would like to attend. The aim is to assign students to as many of these modules as possible. This model considers the total requests:

1 $$\begin{aligned} \max ~ z_1 = \sum _{s \in S} \sum _{k \in K_s^\text {elec}} n_{s,k}. \end{aligned}$$

The model does not force the timetable to be complete (feature 22 in Table 2) so maximising this objective may result in classes with no time or room assignment.

#### Minimise the total number of deviations from mode requests

Students may provide a preference for either the online format or the in-person format. The aim is to align with this preference as much as possible. This model considers the total deviation from mode requests (the amount of mode requests not met):

2 $$\begin{aligned} \min ~ z_2 = \sum _{s \in S} \sum _{c \in C} \tau _{s,c}. \end{aligned}$$

#### Minimise the total number of student scheduling issues

There are two scheduling issues considered in this model. The first is where a student is assigned to two classes that overlap in time. The second is where a student is assigned to two classes that are placed in space and time in such a way that it is impossible to travel between them without leaving one class early or arriving at the other late. This model considers the total number of scheduling issues in the timetable:

3 $$\begin{aligned} \min ~ z_3 = \sum _{s \in S}\sum _{c_1 \in C}\sum _{c_2 \in C} h_{s,c_1,c_2}. \end{aligned}$$

One advantage of having the number of scheduling issues as a soft constraint instead of a hard constraint is that the model is less likely to become infeasible. Another advantage is that this objective gives another measure of solution quality (Barnhart et al., 2022). In a decision-making context, knowing the number of issues is more informative than infeasibility (Sørensen and Dahms, 2014).

There are often so many students at a university that achieving no issues is nearly impossible. The current practice at universities is for students to meet with a staff member and discuss compromising on module choice to resolve scheduling issues.

### Constraints

In this section, the hard constraints of the model are outlined.

#### Linking constraints for resource assignment

It is convenient for the description of the model to be able to switch between the collection of $y^R_{c,r}$ and $y^T_{c,t}$ variables, and the collection of $x_{c,r,t}$ variables. The linking constraints are as follows:

4 $$\begin{aligned} y^R_{c,r}= &  \sum _{t \in T} x_{c,r,t}, ~~ \forall r \in R, c \in C, \end{aligned}$$

5 $$\begin{aligned} y^T_{c,t}\le &  \sum _{r \in R} x_{c,r,t}, ~~ \forall t \in T, c \in C, \end{aligned}$$

6 $$\begin{aligned} \sum _{r \in R} x_{c,r,t}\le &  2y^T_{c,t}, ~~ \forall t \in T, c \in C. \end{aligned}$$

Constraints 4 state that if $y^R_{c,r}$ indicates that a class *c* is happening in a room *r* then this is if and only if exactly one of the $x_{c,r,t}$ variables indicates the same arrangement. Constraints 5 and Constraints 6 combined achieve a similar outcome for time arrangements. Two sets of constraints are needed because the summation in Constraints 5 and Constraints 6 can be equal to two due to how hybrid teaching is modelled in this paper.

#### Classes can only be assigned compatible teaching spaces and timesets

For each $c \in C$ add the following constraints:

7 $$\begin{aligned} \sum _{r \in R} x_{c,r,t}= &  0, ~~ \forall t \in T \setminus T_c, \end{aligned}$$

8 $$\begin{aligned} \sum _{t \in T} x_{c,r,t}= &  0, ~~ \forall r \in R \setminus R_c. \end{aligned}$$

#### Classes should not happen in a teaching space when that space is not available

9 $$\begin{aligned} \sum _{t \in T} \sum _{r \in R} D_0[r,t] x_{c,r,t} = 0, ~~ \forall c \in C. \end{aligned}$$

#### Classes can only be assigned at most one timeset

10 $$\begin{aligned} \sum _{t \in T} y^T_{c,t} \le 1, ~~ \forall c \in C. \end{aligned}$$

#### Classes can only be assigned a maximum of two teaching spaces

11 $$\begin{aligned} \sum _{r \in R\setminus \{r^*\}} y^R_{c,r}\le &  1, ~~ \forall c \in C, \end{aligned}$$

12 $$\begin{aligned} \sum _{r \in R} y^R_{c,r}\le &  2, ~~ \forall c \in C. \end{aligned}$$

Constraints 11 ensure that a class can only be held in at most one in-person teaching space. Constraints 11 and Constraints 12 combined then ensure that if there are two teaching spaces assigned, exactly one will be held in person and the other will be held online.

#### Classes can happen online and in-person if the physical room is appropriate

13 $$\begin{aligned} y^R_{c,r^*} \le 1 - \sum _{r \in R\setminus R^h} y^R_{c,r}, ~~ \forall c \in C. \end{aligned}$$

Constraints 13 ensure that when a class is assigned an in-person teaching space not capable of hybrid teaching then it is impossible for the class to also be assigned the online teaching space and vice versa.

#### In-person classes should not use the same teaching space at the same time

14 $$\begin{aligned} \sum _{c \in R^c_r}\sum _{t \in O_l} x_{c,r,t} \le 1, \quad \forall r \in R\setminus \{r^*\}, l \in L. \end{aligned}$$

#### Module is offered if at least one configuration is offered

15 $$\begin{aligned} g_k |F_k|\ge &  \sum _{f \in F_k} q_{k,f}, ~~ \forall k \in K, \end{aligned}$$

16 $$\begin{aligned} g_k\le &  \sum _{f \in F_k} q_{k,f}, ~~ \forall k \in K. \end{aligned}$$

#### Configuration is offered if and only if every subpart is offered

17 $$\begin{aligned} q_{k,f}|P_{f,k}|= \sum _{p \in P_{f,k}} w_{k,f,p}, ~~ \forall f \in F_k, k \in K. \end{aligned}$$

#### Subpart is offered if at least one class in the subpart is offered

18 $$\begin{aligned} &  w_{k,f,p}|C_{p,f,k}||R||T|\ge \sum _{c\in C_{p,f,k}}\sum _{r \in R}\sum _{t \in T} x_{c,r,t},\nonumber \\ &  \quad \forall p \in P_{f,k}, f \in F_{k}, k \in K, \end{aligned}$$

19 $$\begin{aligned} &  w_{k,f,p} \le \sum _{c\in C_{p,f,k}}\sum _{r \in R}\sum _{t \in T} x_{c,r,t}, \nonumber \\ &  \quad \forall p \in P_{f,k}, f \in F_{k}, k \in K. \end{aligned}$$

#### Staff must be able to attend classes they can teach

For each staff member $h \in H$, let $G = C_h$. For each $(c_1,c_2) \in C_G$ add the following constraints:

20 $$\begin{aligned}&D_2[r_1,r_2,t_1,t_2](x_{c_1,r_1,t_1}+x_{c_2,r_2,t_2}) \le 1,\nonumber \\&\quad \forall t_1 \in T_{c_1},t_2 \in T_{c_2}, r_1 \in R_{c_1}, r_2 \in R_{c_2}. \end{aligned}$$

#### Student does not attend a module they do not request

21 $$\begin{aligned} n_{s,k} \le 0, ~~ \forall k \in K\setminus K_s, s \in S. \end{aligned}$$

#### Student does not attend a module that is not offered

22 $$\begin{aligned} n_{s,k} \le g_k, ~~ \forall k \in K, s \in S. \end{aligned}$$

#### Student must attend all compulsory modules

23 $$\begin{aligned} n_{s,k} = 1, ~~ \forall k \in K_s^\text {core}, s \in S. \end{aligned}$$

#### Student does not attend too many modules

24 $$\begin{aligned} \sum _{k \in K} n_{s,k} \le k^\text {cap}_s, ~~ \forall s \in S. \end{aligned}$$

#### Student does not attend a class that is not offered

25 $$\begin{aligned} \alpha ^{\text {inp}}_{s,c}\le &  \sum _{t \in T}\sum _{r \in R\setminus \{r^*\}} x_{c,r,t}, ~~ \forall c \in C, s \in S, \end{aligned}$$

26 $$\begin{aligned} \alpha ^{\text {onl}}_{s,c}\le &  \sum _{t \in T} x_{c,r^*,t}, ~~ \forall c \in C, s \in S. \end{aligned}$$

#### Student attends a module if they attend a configuration for that module

27 $$\begin{aligned} &  \sum _{f \in F_k} m_{s,k,f} = n_{s,k}, \nonumber \\ &  \quad \forall k \in K, s \in S. \end{aligned}$$

#### Student assigned configuration if they attend a class from each subpart

28 $$\begin{aligned}&\sum _{p \in P_{f,k}} b_{s,k,f,p} = |P_{f,k}|m_{s,k,f},\nonumber \\&\quad ~~ \forall f \in F_k, k \in K, s \in S. \end{aligned}$$

#### Student has at most one class from a subpart

29 $$\begin{aligned}&\sum _{c \in C_{p,f,k}} a_{s,k,f,p,c} = b_{s,k,f,p},\nonumber \\&\quad \forall p \in P_{f,k}, f \in F_k, k \in K, s \in S. \end{aligned}$$

#### Student attends either the online session or the in-person session

30 $$\begin{aligned} a_{s,k,f,p,c}&= \alpha ^{\text {onl}}_{s,c} + \alpha ^{\text {inp}}_{s,c},\nonumber \\&\quad ~~ \forall c \in C_{p,f,k}, p \in P_{f,k}, f \in F_k, k \in K, s \in S. \end{aligned}$$

#### Physical room capacities cannot be exceeded

31 $$\begin{aligned}&\sum _{s \in S_k} \alpha ^{\text {inp}}_{s,c} \le \sum _{r \in R_c\setminus \{r^*\}} cap(r)y^R_{c,r},\nonumber \\&\quad \forall c \in C_{p,f,k}, p \in P_{f,k}, f \in F_k, k \in K. \end{aligned}$$

#### Class subscription capacities cannot be exceeded

32 $$\begin{aligned}&\sum _{s \in S_k} (\alpha ^{\text {inp}}_{s,c} + \alpha ^{\text {onl}}_{s,c}) \le sub(c),\nonumber \\&\quad \forall c \in C_{p,f,k}, p \in P_{f,k}, f \in F_k, k \in K. \end{aligned}$$

#### Parent–child classes

It is often the case that some classes are prerequisites for other classes. For example, to attend a workshop in a module the student should attend the lecture for that module also. Given a student $s \in S$, for every parent/child class pair (with the child class denoted as $c_{ch}$ and the parent denoted as $c_{par}$) add the following constraint:

33 $$\begin{aligned} \alpha ^{\text {inp}}_{s,c_{ch}} + \alpha ^{\text {onl}}_{s,c_{ch}} \le \alpha ^{\text {inp}}_{s,c_{par}} + \alpha ^{\text {onl}}_{s,c_{par}}. \end{aligned}$$

#### Mode requests

The $\tau$ variables need to be linked to the allocation of students.

34 $$\begin{aligned} \tau _{s,c}\ge &  \pi _s\left( \alpha _{s,c}^{\text {onl}} - \alpha _{s,c}^{\text {inp}} \right) , ~~ \forall s \in S, c \in C, \end{aligned}$$

35 $$\begin{aligned} \tau _{s,c}\le &  \alpha _{s,c}^{\text {onl}} + \alpha _{s,c}^{\text {inp}}, ~~ \forall s \in S, c \in C. \end{aligned}$$

Constraints 34 force the value to one if the preference is not met and Constraints 35 force the value to zero if the class is not attended by that student.

#### Detect if a student has an overlapping class

For the objective $z_3$ described in Eq. 3 to correctly detect overlaps, the $h_{s,c_1,c_2}$ variables need to be linked to the assignment of student $s \in S$. This is done by first adding the following constraints:

36 $$\begin{aligned} \alpha _{s,c}^\text {inp} \times y_{c,t}^T= &  \beta _{s,c,t}^\text {inp}, ~~ \forall s \in S, c \in C, t \in T, \end{aligned}$$

37 $$\begin{aligned} \alpha _{s,c}^\text {onl} \times y_{c,t}^T= &  \beta _{s,c,t}^\text {onl}, ~~ \forall s \in S, c \in C, t \in T. \end{aligned}$$

Next, for every student $s \in S$ and each $(c_1,c_2) \in C_{C_s}$, where $C_{C_s}$ is the pair-wise combinations of classes that the student can take, the following constraints are added:

38 $$\begin{aligned}&D_1[t_1,t_2](\beta ^{\text {onl}}_{s,c_1,t_1}+\beta ^{\text {inp}}_{s,c_1,t_1}+ \beta ^{\text {onl}}_{s,c_2,t_2}+\beta ^{\text {inp}}_{s,c_2,t_2})\nonumber \\&\quad \le 1 + h_{s,c_1,c_2}, ~~ \forall t_1 \in T_{c_1},t_2 \in T_{c_2}. \end{aligned}$$

Constraints 36 to 38 ensure that $h_{s,c_1,c_2}$ is equal to one if $c_1$ and $c_2$ overlap in time and student *s* is assigned to both of classes. Constraints 36 and 37 are nonlinear but this can be resolved by replacing them with the following constraints:

39 $$\begin{aligned} &  \beta ^{\text {inp}}_{s,c,t} \le \alpha ^{\text {inp}}_{s,c}, ~~ \forall s \in S, c \in C, t \in T, \end{aligned}$$

40 $$\begin{aligned} &  \beta ^{\text {onl}}_{s,c,t} \le \alpha ^{\text {onl}}_{s,c}, ~~ \forall s \in S, c \in C, t \in T, \end{aligned}$$

41 $$\begin{aligned} &  \beta ^{\text {inp}}_{s,c,t} \le y^T_{c,t}, ~~ \forall s \in S, c \in C, t \in T, \end{aligned}$$

42 $$\begin{aligned} &  \beta ^{\text {onl}}_{s,c,t} \le y^T_{c,t}, ~~ \forall s \in S, c \in C, t \in T, \end{aligned}$$

43 $$\begin{aligned} &  \beta ^{\text {inp}}_{s,c,t} \ge \alpha ^{\text {inp}}_{s,c} + y^T_{c,t} - 1, ~~ \forall s \in S, c \in C, t \in T, \end{aligned}$$

44 $$\begin{aligned} &  \beta ^{\text {onl}}_{s,c,t} \ge \alpha ^{\text {onl}}_{s,c} + y^T_{c,t} - 1, ~~ \forall s \in S, c \in C, t \in T. \end{aligned}$$

#### Detect if a student has enough travel time between classes

For the objective $z_3$ described in Eq. 3 to correctly detect travel time issues, the $h_{s,c_1,c_2}$ variables again need to be linked to the assignment of student $s \in S$. This is done in a similar way to overlap detection and involves first adding the following constraints:

45 $$\begin{aligned} \beta ^{\text {inp}}_{s,c,t} \times y^R_{c,r}= &  \gamma _{s,c,r,t},\nonumber \\ &  \quad \forall s \in S, c \in C, t \in T, r \in R \setminus \{ r^*\}, \end{aligned}$$

46 $$\begin{aligned} \beta ^{\text {onl}}_{s,c,t} \times y^R_{c,r^*}= &  \gamma _{s,c,r^*,t},\nonumber \\ &  \quad \forall s \in S, c \in C, t \in T. \end{aligned}$$

Then, for every student $s \in S$ and each $(c_1,c_2) \in C_{C_s}$, where $C_{C_s}$ is the pair-wise combinations of classes that the student can take, add the following constraints:

47 $$\begin{aligned} &  D_2[r_1,r_2,t_1,t_2](\gamma _{s,c_1,r_1,t_1}+\gamma _{s,c_2,r_2,t_2})\nonumber \\ &  \quad \le 1 + h_{s,c_1,c_2},\nonumber \\ &  \quad \forall t_1 \in T_{c_1},t_2 \in T_{c_2}, r_1 \in R_{c_1}, r_2 \in R_{c_2}. \end{aligned}$$

These constraints ensure that $h_{s,c_1,c_2}$ is equal to one if student *s* does not have enough time to travel between classes $c_1$ and $c_2$. Once again these are nonlinear constraints so Constraints 45 and Constraints 46 are replaced with the following:

48 $$\begin{aligned} &  \gamma _{s,c,r,t} \le \beta ^{\text {inp}}_{s,c,t},\nonumber \\ &  \forall s \in S, c \in C, t \in T, r \in R \setminus \{ r^*\}, \end{aligned}$$

49 $$\begin{aligned} &  \gamma _{s,c,r,t} \le y^R_{c,r},\nonumber \\ &  \forall s \in S, c \in C, t \in T, r \in R \setminus \{ r^*\}, \end{aligned}$$

50 $$\begin{aligned} &  \gamma _{s,c,r,t} \ge \beta ^{\text {inp}}_{s,c,t} + y^R_{c,r} - 1,\nonumber \\ &  \forall s \in S, c \in C, t \in T, r \in R \setminus \{ r^*\}, \end{aligned}$$

51 $$\begin{aligned} &  \gamma _{s,c,r^*,t} \le \beta ^{\text {onl}}_{s,c,t}, ~~ \forall s \in S, c \in C, t \in T,\end{aligned}$$

52 $$\begin{aligned} &  \gamma _{s,c,r^*,t} \le y^R_{c,r^*}, ~~ \forall s \in S, c \in C, t \in T, \end{aligned}$$

53 $$\begin{aligned} &  \gamma _{s,c,r^*,t} \ge \beta ^{\text {onl}}_{s,c,t} + y^R_{c,r^*} - 1,\nonumber \\ &  \quad \forall s \in S, c \in C, t \in T. \end{aligned}$$

## Solution method

The solution method used in this paper involves a preprocessing stage and then a stage that solves the three objectives in a certain order.

### Preprocessing steps

The full mathematical formulation includes some variables and constraints that are not necessary and therefore can be removed from the model. In this section, some of the steps taken to remove redundant variables and constraints are outlined. This does not necessarily remove all redundancies and whilst more sophisticated reduction methods exist (see Holm et al., 2022) these are not employed in this paper.

#### Variables that can be removed

The first collection of variables that can be omitted from the model includes $x_{c,r,t}$ variables for each $c \in C$ where either $t \in T{\setminus } T_c$ or $r \in R{\setminus } R_c$. This is because Constraints 7 and 8 force these variables to be zero. Similarly, if $D_0[r,t] = 1$ for some pairing (*r*, *t*) where $t \in T$ and $r \in R$ then for all classes $c \in C$ it is the case that Constraint 9 is forcing $x_{c,r,t} = 0$ and therefore these variables can be removed from the model.

The second collection of variables that can be omitted are from the student sectioning part of the model. $n_{s,k}=0$ for $s \in S$ and $k \in K\setminus K_s$ therefore these variables can be removed. Furthermore, any variable with $k \in K\setminus K_s$ in the indexing for a given student $s \in S$ can be removed as these will be forced to zero. Finally, any variable with $c \in C_{p,f,k}$ for $k \in K\setminus K_s$ in the indexing can be removed. Essentially what is meant by this is that any variable relating to a course a student does not want to attend is removed from the model.

#### Overlap and travel time constraints

For any two timesets $t_1 \in T$ and $t_2 \in T$, it is always true that $D_1[t_1,t_2] \le D_2[r_1,r_2,t_1,t_2]$ for any choice of $r_1$ and $r_2$ from the set *R*. Therefore if two timesets that overlap are identified and Constraints 38 to 44 are included in the model there is no need to include Constraints 47 to 53 for those two timesets. There is a similar process for Constraints 20 where if two timesets overlap there is no need to include the constraint for every room combination. We only need to constrain the time assignment using the $y^T$ variables in this case.

Furthermore, when considering a pair of classes $c_1$ and $c_2$, it is possible that for a pair of timesets $(t_1,t_2) \in T_{c_1} \times T_{c_2}$ we have that $D_2[r_1,r_2,t_1,t_2] = 0$ for any pair of rooms $(r_1,r_2) \in R_{c_1} \times R_{c_2}$. This means there is no need to include Constraints 47 to 53 for this timeset pair (for this pair of classes). These two checks help reduce the total number of constraints, especially if classes have a large number of teaching spaces they could be assigned to.

A final check that is employed is we do not check for scheduling issues for pairs of classes that we know cannot be attended together. For example, students can only participate in one class from a subpart (Constraint 29), therefore, we will never have problems with a pair of classes from the same subpart. The impact of this preprocessing step is dependent on the course and module structure assumed.

### Objective ordering and solving

The solution method involves solving three single objective problems sequentially. The objectives are ordered based on importance. One such ordering is as follows:

1. Maximise the total number of elective module requests met ($z_1$).
2. Minimise the total number of deviations from mode requests ($z_2$).
3. Minimise the total number of student scheduling issues ($z_3$).

In this case, the approach would be to first maximise $z_1$ using a commercial solver. Denote the value of this solution as $z_1^*$. The constraint $z_1 = z_1^*$ is added and the model is solved to maximise $z_2$. Similarly, denote the value of this solution as $z_2^*$. We add the constraint $z_2 = z_2^*$ to the model and minimise $z_3$.

## Computational experiments

In this section, the experimental data used to test the model is described and computational experiments are performed to demonstrate the use of the model. All experiments were completed on an internal computing node running Ubuntu 22.04.1 LTS with an Intel Xeon Gold 6348 CPU running at 2.60GHz and 528GB of RAM. The model was implemented in Python 3.9.16 and solutions were found using Gurobi Optimizer version 10.0.2.

### Experimental data

The experimental data has been taken from the ITC-2019 competition (Müller et al., 2018). These data sets are based on data taken from real-world universities. These provide the model with most of the information needed. However, since these data sets do not explicitly consider the online space, there are features of the model presented in this paper that require specifying for each experiment. This includes:

- The value of $d^*$ in the matrix *A*.
- The value of $\pi _s$ for each student $s \in S$.
- The value of $k^\text {cap}_s$ for each student $s \in S$.
- The set $R^h$ that identifies which physical rooms are suitable for hybrid teaching.

In our experiments, we assume that $d^*$ is one and a half times bigger than the largest distance between physical rooms. We chose this number as it roughly estimates the travel time from student accommodation to campus for some universities in the UK we are familiar with. In practice, this value will vary depending on the university. Setting the distance to this value also makes switching between modes undesirable.

To align the data with the motivation for this model, we are also creating a shortage of capacity in physical rooms. This shortage reflects a situation similar to that of the COVID-19 pandemic. Barnhart et al. (2022) quoted that during this period they had a fourfold reduction in physical space on campus and therefore we are reducing the capacity of each room in the data sets by 75%. Applying this reduction to the four instances means that room capacity is the limiting factor to the total in-person attendance.

It is also assumed that every student has one of three preferences: online teaching, in-person teaching or no preference ($\pi _s$ equal to $-1$, 1 or 0 respectively). A systematic way of modifying the data is applied. In the ITC-2019 data, each student has an “ID” number. If a student’s ID number is a multiple of three, they prefer the online mode. If the remainder after dividing their ID number by three is two, they have no preference. All other students prefer the in-person mode.

In the ITC-2019 competition, students are assigned to every module they request. This is equivalent to treating every module as compulsory. To demonstrate the objective of maximising the number of elective module requests met, we assume in our experiments that all modules are elective. This means there is no requirement to assign a student to any of the modules they request. The value of $k^\text {cap}_s$ for each $s \in S$ is the length of the list of requests for that student.

Each “SameAttendee” distribution constraint used to model staff in the ITC-2019 problem has an associated list of classes (see Müller et al., 2018). The class lists from the required distributions of this type in the data are used as part of our staff constraints. In particular, each distribution constraint in the data is a staff member $h \in H$ and the class list forms the set $C_h$ used in Constraints 20.

Finally, we need to specify the set $R^h$. It is assumed that a class can only happen in the hybrid mode if the physical room assigned to it has a capacity of 30 or more people. In particular, if $cap(r) \ge 30$ for $r \in R$ then $r \in R^h$. The assumption is based on the observation that only the larger lecture halls at universities have the correct audio-visual equipment for hybrid teaching.

The four ITC-2019 instances used in this paper are from various stages of the competition and are currently archived on the competition website. Table 4 records the name of each instance and the critical features of that instance. To reduce the size of the problem, a subset of students from each instance is used in the experiment. This is defined by the ID of the first student in the subset and how many students are taken after that student. For example, with the instance *mary-fal18*, Table 4 says that the “Start” is equal to 600 and “Count” is equal to 400 meaning only students 600 to 999 are considered.

**Table 4:** Instances from the ITC-2019 with their key features. “Start” and “Count” indicate the subset of students used in the experiment

Instance	$|S |$	$|K |$	$|C |$	$|T |$	$|R |$	$|R^h \setminus \{ r^*\} |$	Start	Count
wbg-fal10	19	21	150	154	8	0	1	19
pu-cs-fal07	2002	44	174	182	14	4	1000	1000
muni-fsps-spr17	865	226	561	1953	45	40	1	100
mary-fal18	5051	540	951	503	94	35	600	400

Column $|R^h \setminus \{ r^*\} |$ is based on our assumptions about hybrid rooms

#### Instance one: wbg-fal10

It is possible to solve any ordering of objectives for *wbg-fal10* as it is a small instance. Table 5 provides the objective values for the six possible orderings. It can be seen that only $z_1$ (the number of elective module requests met) and $z_2$ (the number of mode request deviations) influence each other. Table 6 shows that lexicographically optimal solutions for this instance either meet all the student mode preferences or offer all requested modules.

#### Instance two: pu-cs-fal07

In the instance *pu-cs-fal07*, 1000 students are considered. Like *wbg-fal10*, no student conflicts arise in this instance and the only objectives that appear to influence each other are $z_1$ and $z_2$. It can be seen from Table 5 that the lexicographically optimal solutions have one of two objective values. Looking at the class attendance, we see that the ratio of in-person to online attendance is about 1:2 suggesting that when students have no preference for a mode they get placed into the online mode.

#### Instance three: muni-fsps-spr17

Only 100 students from *muni-fsps-spr17* are used, however, each student attends nearly eight times the number of classes on average than in *pu-cs-fal07*. When $z_1$ is prioritised over $z_2$, there is a larger increase in $z_2$ than in *pu-cs-fal07*. This suggests a stronger link between the two objectives in this instance than in *pu-cs-fal07*. There is some relationship between $z_2$ and $z_3$ also.

#### Instance four: mary-fal18

The *mary-fal18* instance considers 400 students. Table 5 shows that the lexicographically optimal solutions have one of five objective values with only six possible orderings. That makes this instance a good instance to demonstrate how objective ordering can influence the solution.

Orderings prioritising $z_1$ lead to solutions with the maximum amount of module requests met. The best possible values of $z_2$ and $z_3$ can be attained if we optimise those first (in either order), however when $z_1$ is optimised before one or both of these objectives this cannot be done. This shows that $z_1$ influences objectives directly and influences relations between objectives (compare orderings $z_1,z_2,z_3$ with $z_1,z_3,z_2$).

**Fig. 2:**
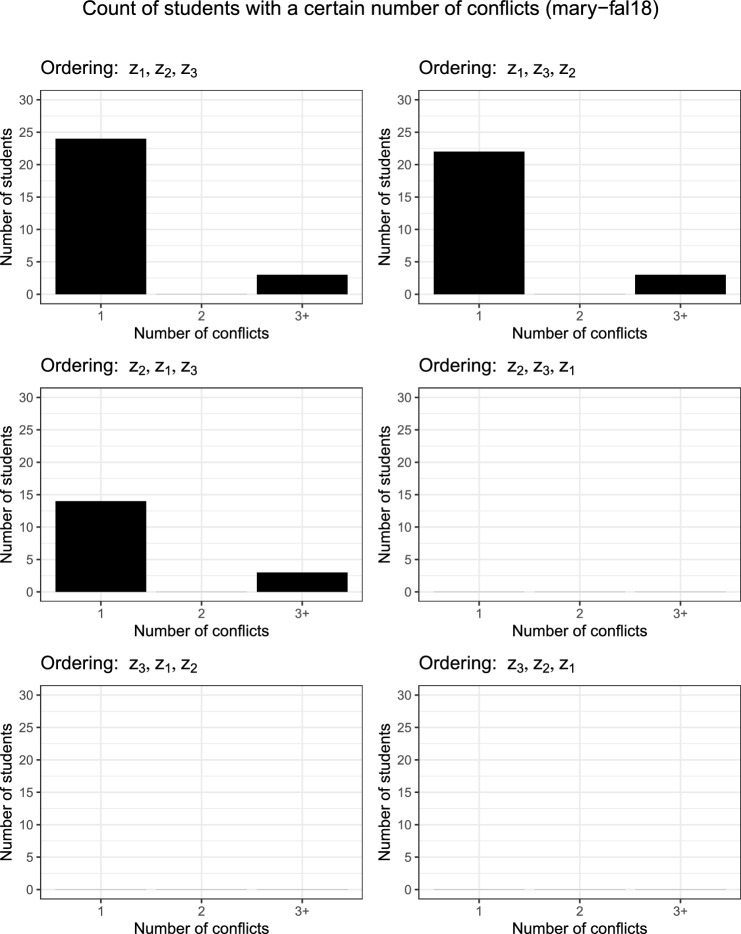
Count of students who experience one, two or three plus conflicts with their timetable. The majority of students do not experience any conflicts therefore the zero bar is omitted

**Fig. 3:**
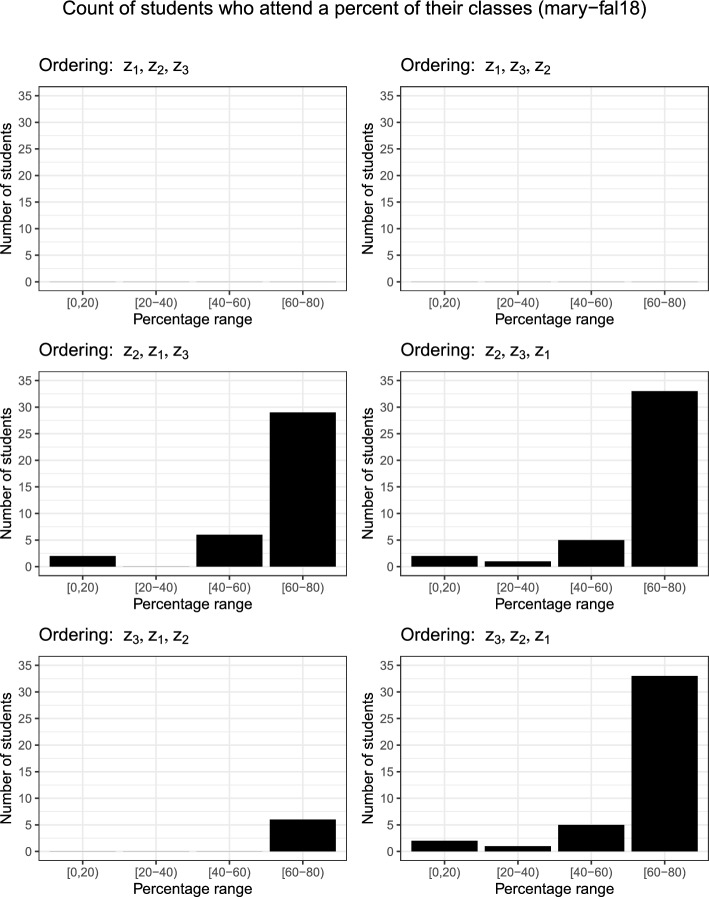
Count of students who have a certain percentage of their classes met. They are aggregated into ranges so that the bars are an appropriate size. The [90, 100] range is omitted but contains the remaining students

Figure 3 shows that if matching the mode preference ($z_2$) is prioritised over module requests ($z_1$) then fewer requests can be met. Figure 2 shows clearly that prioritising $z_3$ over $z_1$ removes conflicts but also shows that matching students’ mode requests can reduce the number of conflicts (likely due to its direct influence on the number of modules attended by students indicated by Fig. 3).

**Table 5:** Objective values for each instance and their six potential objective orderings

Ordering	Objective			Classes attended			Switch
	$z_1$	$z_2$	$z_3$	Total	In-person	Online	$z_4$
wbg-fal10							
$z_1$,$z_2$,$z_3$	97	43	0	199	27	172	5
$z_1$,$z_3$,$z_2$	97	43	0	199	27	172	5
$z_2$,$z_1$,$z_3$	71	0	0	142	16	126	1
$z_2$,$z_3$,$z_1$	71	0	0	142	17	125	2
$z_3$,$z_1$,$z_2$	97	43	0	199	27	172	6
$z_3$,$z_2$,$z_1$	71	0	0	142	17	125	2
pu-cs-fal07							
$z_1$,$z_2$,$z_3$	1226	11	0	1611	567	1044	0
$z_1$,$z_3$,$z_2$	1226	11	0	1611	596	1015	0
$z_2$,$z_1$,$z_3$	1220	0	0	1599	665	934	0
$z_2$,$z_3$,$z_1$	1220	0	0	1599	641	958	0
$z_3$,$z_1$,$z_2$	1226	11	0	1611	561	1050	0
$z_3$,$z_2$,$z_1$	1220	0	0	1599	641	958	0
muni-fsps-spr17							
$z_1$,$z_2$,$z_3$	980	106	8	1552	567	985	13
$z_1$,$z_3$,$z_2$	980	114	0	1552	535	1017	11
$z_2$,$z_1$,$z_3$	926	0	0	1366	503	863	15
$z_2$,$z_3$,$z_1$	926	0	0	1366	450	916	8
$z_3$,$z_1$,$z_2$	980	114	0	1552	562	990	11
$z_3$,$z_2$,$z_1$	926	0	0	1366	450	916	8
mary-fal18							
$z_1$,$z_2$,$z_3$	1597	109	36	1613	517	1096	13
$z_1$,$z_3$,$z_2$	1597	111	34	1613	512	1101	13
$z_2$,$z_1$,$z_3$	1498	0	26	1509	536	973	2
$z_2$,$z_3$,$z_1$	1480	0	0	1480	544	936	5
$z_3$,$z_1$,$z_2$	1571	98	0	1571	514	1057	13
$z_3$,$z_2$,$z_1$	1480	0	0	1480	544	936	5

$z_4$ is the number of students who switch between online and in-person classes twice or more on the same day (measured using the found solution, not optimised)

**Table 6:** Key metrics for individual students in tabular form

Ordering	% Electives			# Conflicts			% Mode		
	Max	Min	Avg	Max	Min	Avg	Max	Min	Avg
wbg-fal10									
$z_1$,$z_2$,$z_3$	100	100	100.00	0	0	0.00	100	25	77.42
$z_1$,$z_3$,$z_2$	100	100	100.00	0	0	0.00	100	30.77	77.93
$z_2$,$z_1$,$z_3$	100	0	73.51	0	0	0.00	100	100	100.00
$z_2$,$z_3$,$z_1$	100	0	73.51	0	0	0.00	100	100	100.00
$z_3$,$z_1$,$z_2$	100	100	100.00	0	0	0.00	100	25	77.90
$z_3$,$z_2$,$z_1$	100	0	73.51	0	0	0.00	100	100	100.00
pu-cs-fal07									
$z_1$,$z_2$,$z_3$	100	100	100.00	0	0	0.00	100	0	99.66
$z_1$,$z_3$,$z_2$	100	100	100.00	0	0	0.00	100	0	99.66
$z_2$,$z_1$,$z_3$	100	0	99.67	0	0	0.00	100	100	100.00
$z_2$,$z_3$,$z_1$	100	50	99.70	0	0	0.00	100	100	100.00
$z_3$,$z_1$,$z_2$	100	100	100.00	0	0	0.00	100	0	99.66
$z_3$,$z_2$,$z_1$	100	50	99.70	0	0	0.00	100	100	100.00
muni-fsps-spr17									
$z_1$,$z_2$,$z_3$	100	100	100.00	1	0	0.08	100	56.52	94.97
$z_1$,$z_3$,$z_2$	100	100	100.00	0	0	0.00	100	52.17	94.62
$z_2$,$z_1$,$z_3$	100	73.33	95.75	0	0	0.00	100	100	100.00
$z_2$,$z_3$,$z_1$	100	73.33	95.75	0	0	0.00	100	100	100.00
$z_3$,$z_1$,$z_2$	100	100	100.00	0	0	0.00	100	52.17	94.62
$z_3$,$z_2$,$z_1$	100	73.33	95.75	0	0	0.00	100	100	100.00
mary-fal18									
$z_1$,$z_2$,$z_3$	100	100	100.00	6	0	0.09	100	0	94.62
$z_1$,$z_3$,$z_2$	100	100	100.00	6	0	0.09	100	0	94.54
$z_2$,$z_1$,$z_3$	100	0	95.08	6	0	0.07	100	100	100.00
$z_2$,$z_3$,$z_1$	100	0	94.36	0	0	0.00	100	100	100.00
$z_3$,$z_1$,$z_2$	100	66.67	98.83	0	0	0.00	100	0	94.96
$z_3$,$z_2$,$z_1$	100	0	94.36	0	0	0.00	100	100	100.00

“% Electives” is the percentage of elective modules a student is assigned from their list. “# Conflicts” represents the number of scheduling issues a student has. “% Mode” is the percentage of classes a student is assigned that is in their preferred mode

## Analysis and extensions

We believe that considering the hybrid teaching format presents new challenges that have not been fully researched in the timetabling literature. Some of these challenges are discussed here.

### Impact of including hybrid teaching

From Table 5, it can be seen that many classes are attended online. This demonstrates how well the model presented in this paper satisfies the mode preferences. By construction, one-third of the student population in the instances preferred in-person teaching and the other two-thirds either had no preference or preferred online teaching. This is roughly represented in the ratio of classes attended in-person to classes attended online. The results in the previous section also suggest that the model takes advantage of the students who have no preference and assigns them to the more flexible and high-capacity online space. This alleviates pressure on physical resources whilst delivering the same amount of teaching. From a modelling point of view, the inclusion of hybrid teaching adds more flexibility to the timetabling process because a purely online timetable is not impacted by travel times and room capacity in the same way as a purely in-person timetable. In practice, it would be up to a practitioner to decide to what extent the online space should be used.

### Impact of objective ordering

If a university has preconceived thoughts on how the objectives should be ordered, then this model can cater to those preferences. This model can also be used to gain more information about the problem. Trying different orders of objectives can be used to gain insight into how and to what extent the objectives influence each other.

One example is that in the instance *mary-fal18*. It was identified here that different orderings typically resulted in differing solutions. A university may have an idea of an objective order they want but find that a different ordering is better suited to their needs.

Table 5 shows that differences in the solutions generated by different orderings might be very different or only have subtle differences. This demonstrates that whilst the ordering of objectives does impact the difference in solutions, it seems that the extent of the difference is unique for each instance.

### Model extensions

#### Compulsory modules and credit loads

Constraints 23 describe compulsory modules. The model becomes infeasible if a student cannot be offered a mandatory module. This is not a useful finding in practice as universities ultimately need a timetable (Sørensen and Dahms, 2014). One approach would be to relax the constraint and instead attach a high penalty for not ensuring every student is assigned to their mandatory modules. Including hybrid teaching offers flexibility as it facilitates a meaningful two-stage approach of time assignment and then room assignment (Barnhart et al., 2022).

It is common for a student to not only have mandatory or compulsory modules but to be required to attend a certain total number of modules making up a credit load. The model presented in this paper could be extended so that students are assigned enough modules to meet a specific credit load. This would require students to provide an extended list of elective modules so there are sufficient options.

#### Fairness and extended preferences

All the objective functions present in this paper are aggregate measures so there is no concept of “fairness” when assigning students. For example, if two students request two modules then a solution where one student is assigned to two modules and the other is assigned to none is equivalent in objective value to a solution where both students are assigned to one module each. The latter solution is arguably “fairer”.

A similar situation can happen with preferences. It is possible that a solution could assign students so that some students attend the mode they prefer for every class whilst some students attend none of their classes in their preferred mode. Currently, each student has a mode preference for all modules. The model could be extended to consider a student’s mode preferences for each module or even mode preferences for each class. This extension makes the problem more true to life but exacerbates the issues surrounding fair assignments.

#### Controlling mode of attendance

Universities may want control over what mode students study in. The mode that students attend their classes relates heavily to where students choose to live, how busy the campus is, and how resources are used on campus at the expense of the university.

For example, if a university has on-site student accommodation they may aim to have a minimum percentage of classes attended in-person. A university would do this to make renting this accommodation a more appealing choice for students. On the other hand, a university may wish to limit the percentage of teaching done in person. This may be to reduce student density on campus, either to reduce the spread of infection or to reduce the amount of energy and money spent on lighting and heating buildings at the university.

More parameters and constraints would need to be introduced to model this extension but it is useful for a practitioner to investigate the effect of changing these parameters.

#### Switching between modes of study

A timetable where students and staff make multiple switches between modes on the same day is undesirable. Multiple switches could mean more traffic on and around the university which has both a health and environmental impact (potential spread of disease and increase in vehicle emissions). Whilst many students only attend a single mode of study or switch between the two modes only once, Table 5 shows that without any optimisation there are students that switch between modes twice or more on the same day. An extension to this model would be to have an objective to minimise the number of switches that each student makes in a working day. However, research currently underway indicates that solving this problem is computationally challenging.

### Solution method improvements

As the focus of this paper was the explicit modelling of the hybrid teaching feature of UCTTP, we utilised an exact approach with small instances to test the model and demonstrate what happens to the solution if objectives are reordered. This method is not an efficient way of solving this problem and better methods could be applied. For example, the winner of the ITC-2019 uses a variety of techniques to find solutions to a similar problem (Mikkelsen and Holm, 2022). Other methods applied to similar problems have involved local searches and simulated annealing (Babaei et al., 2015). We are currently developing a method to find solutions for particular orderings more efficiently and a method to explore trade-offs between objectives. The exact solutions reported in this work will be used to assess how well this method performs.

## Conclusion

Several gaps in the university module timetabling literature are identified at the beginning of this paper. To address these gaps, this paper presents a formal and mathematical description of a multi-objective post-enrolment timetabling model with student sectioning and considerations for hybrid teaching. The novel feature of this work is the explicit modelling of hybrid classes, which have an in-person and online element occurring at the same time. The assignment of students in this model is demand-driven. Individual students request modules they want to take and the model creates a timetable that tries to satisfy these requests. It also tries to ensure students attend their teaching in the mode that they prefer. This is demonstrated in a series of computational experiments using modified benchmark data from the 2019 International Timetabling Competition. The modifications are so we can demonstrate the novel features under a setting where these features would be most relevant (for example, severely reduced capacity). In these experiments, an exact lexicographic solution method is used to show that solutions to this model depend on both the input data and the ordering of the objectives. This observation shows that the model can be used by practitioners who have different strategic priorities and are based at different universities. Finally, a list of potential extensions to this model is presented. Items on this list are starting points for future research. Exploring these extensions would benefit both the timetabling community and the wider large-scale integer optimisation community.
